# Neighborhood disadvantage and brain myelination: Insights from infancy to childhood

**DOI:** 10.1162/IMAG.a.1288

**Published:** 2026-06-25

**Authors:** Leela Shah, Elizabeth Bond, Elizabeth Planalp, Marissa DiPiero, Jayse Weaver, Irene Quiterio Perez, Kuan-Fu Chen, Corrina Frye, Steven Kecskemeti, Andrew Alexander, Douglas Dean

**Affiliations:** Waisman Center, University of Wisconsin–Madison, Madison, WI, United States; Neuroscience Training Program, University of Wisconsin–Madison, Madison, WI, United States; Medical Physics, University of Wisconsin–Madison, Madison, WI, United States; Psychiatry, University of Wisconsin–Madison, Madison, WI, United States; Pediatrics, University of Wisconsin–Madison, Madison, WI, United States

**Keywords:** early life adversity, neighborhood disadvantage, area deprivation index, myelin, T1 relaxometry, brain development

## Abstract

Early life adversity (ELA), including poverty and socioeconomic disadvantage, can negatively impact neurodevelopment and long-term health outcomes. Here, we examined associations between neighborhood-level disadvantage, indexed by the Area Deprivation Index (ADI), and white matter myelination in infants and children aged 0–10 years (n = 61 MRI acquisitions across 43 children). Quantitative MRI-derived longitudinal relaxation rates (R1) are sensitive to myelin content and were used to assess white matter development. Higher ADI national percentiles (reflecting greater neighborhood disadvantage) were associated with lower R1 in several white matter regions, including the internal capsule, corona radiata, cingulate gyrus, fronto-occipital fasciculus, superior longitudinal fasciculus, and uncinate fasciculus. Whole-brain voxel-wise analyses further supported negative associations between ADI and myelination. These findings were stronger in the infant subsample and implicate white matter pathways supporting cognitive, socioemotional, and motor functions, suggesting that early environmental disadvantage may influence neurodevelopmental trajectories. Future work should examine which components of ADI drive these associations and how resilience may buffer the effects of disadvantage.

## 1 Introduction

From late gestation through early childhood, the brain undergoes rapid development and is particularly sensitive to environmental influences (Hodel, 2018; Tau & Peterson, 2010). During this critical window, both biological and psychosocial stressors can disrupt developmental trajectories, increasing the risk of adverse cognitive, physical, and mental health outcomes (Farooq et al., 2024; Flouri et al., 2010; Nelson et al., 2020). Environmental influences, such as household stability, neighborhood safety, and access to socioemotional and cognitive support, play a pivotal role in shaping developmental trajectories (Minh et al., 2017), and adverse exposures such as poverty, environmental toxins, and family instability can increase vulnerability to poor cognitive and socioemotional outcomes (National Research Council & Institute of Medicine, 2004). In addition, socioeconomic disadvantage has been associated with delays in language (Brandes-Aitken et al., 2019), numerical understanding (Short & McLean, 2023), executive function (Fitzpatrick et al., 2014), and socioemotional development (Javanbakht et al., 2016), as well as long-term reductions in goal persistence and career aspirations (Hsieh & Huang, 2014; Hu et al., 2022). Thus, understanding the complex interplay of negative and positive environmental exposures on child neurodevelopment is important to promote long-term positive outcomes and inform targeted interventions and policies.

Early life adversity (ELA) refers to sources of disadvantage in childhood and adolescence that deviate from expected norms and require significant behavioral, neurological, or psychological adaptation (Duffy et al., 2018). An estimated 40–50% of children worldwide experience at least one form of ELA, including abuse, neglect, violence, or economic hardship (Duffy et al., 2018; Green et al., 2010). Although poverty is a key driver of ELA, recent frameworks emphasize that poverty itself is not the monolithic predictor of risk, but that children growing up in poverty may also experience other disadvantages (DeJoseph et al., 2024). Families from low-income and racially minoritized backgrounds are more likely to live in communities with limited access to various protective factors, including but not limited to safe housing, quality education, healthcare, and nutritious food. The absence of such positive experiences can contribute to chronic stress and reduced access to developmental resources for children (DeJoseph et al., 2024). Because risk is shaped by interrelated adverse and protective influences at both the individual and macro level, measures that capture contextual adversity are critical for identifying environmental factors that may influence development. The Area Deprivation Index (ADI) is one such measure, offering nuanced, census-block-level quantification of neighborhood disadvantage in the United States through indicators of income, education, housing quality, and employment (Kind & Buckingham, 2018; Kind et al., 2014; University of Wisconsin School of Medicine Public Health, n.d.; Zuelsdorff et al., 2020). Higher ADI national percentiles are indicative of greater neighborhood disadvantage and have been associated with lower cognitive performance and socioemotional differences in children, including greater psychopathology and diminished vocabulary development (Flouri et al., 2010; Hooper et al., 2022; Jones & Shen, 2014).

Infancy is a period of rapid development, offering a salient window during which environmental disadvantage may be associated with alterations in neurodevelopmental trajectories. During this time, the brain is rapidly forming, strengthening, and pruning synaptic connections in response to environmental input; adverse environments may influence these experience-dependent processes, potentially shaping neural circuits that underlie cognitive emotional and behavioral functioning across the lifespan (Milbocker et al., 2021; Tierney & Nelson, 2009). Prior work has demonstrated that childhood adversity is linked to lower white matter microstructure assessed by diffusion MRI and lower cortical volume in regions related to executive function, memory, and emotion regulation, including the corpus callosum, corona radiata, uncinate fasciculus, cingulum, dorsolateral prefrontal cortex, and hippocampus (Ku et al., 2024; McCarthy-Jones et al., 2018; Ugwu et al., 2015). However, these studies rely on retrospective reports and assess brain structure in adolescence or adulthood, missing the critical early period of development during infancy and early childhood when brain development is most rapid (Dean et al., 2015; Matthews et al., 2018). Recent diffusion and functional MRI evidence suggests that neighborhood-level disadvantage during pregnancy and early infancy, captured through measures such as ADI or the Social Vulnerability Index, is associated with differences in microstructural brain development in the cingulum, uncinate fasciculus, and fornix, and with connectivity across the brain as early as the fetal and neonatal stages (Cook et al., 2024; Lean et al., 2022). While adversity metrics have been correlated with measures of brain volume and white matter microstructure, no work to our knowledge has examined associations between neighborhood disadvantage and brain development using measures sensitive to myelin content. Investigating links between environmental disadvantage and brain myelin content is critical given the crucial role myelin plays in propagating signals across the brain, ultimately supporting long-term processing speed and cognitive ability across developmental domains (Beaulieu et al., 2020; Corrigan et al., 2022; Fornari et al., 2007). The first decade of life is characterized by rapid and regionally specific increases in myelin, which closely track emerging motor, language, cognitive, and socioemotional abilities, further emphasizing the importance of studying myelin development during infancy and early childhood (Abel et al., 2020; Beaulieu et al., 2020; Corrigan et al., 2022; S. Deoni et al., 2018). Additionally, myelin is sensitive to external influences such as nutrition, stress, and environmental enrichment (Forbes & Gallo, 2017) and is associated with developmental outcomes and functional ability throughout the lifespan (Abel et al., 2020; Beaulieu et al., 2020; Corrigan et al., 2022; S. Deoni et al., 2018), making it a key biological substrate through which environmental disadvantage may impact neurodevelopment.

Based on the literature outlined above, this study considers how neighborhood disadvantage may be associated with neurodevelopment in a sample of infants and elementary-aged children. We hypothesized that higher neighborhood disadvantage would be associated with reduced myelination in white matter regions critical to cognitive and emotional development. By focusing on early developmental stages, including infancy, this study aims to improve understanding of how contextual disadvantage is associated with early brain development and to assess whether such associations are detectable during early developmental windows.

## 2 Methods

### 2.1 Participants

Data were collected from 43 children aged 3.0 months to 10.9 years. Children were recruited from the greater Madison, WI, area between 2019 and 2024. Demographics are included in the table below (Table 1), and demographics by cohort are included in Supplementary Table S1. The composite sample includes two sub-samples, the first including children recruited at birth who completed up to three MRI sessions across the first 3 years of life, and the second including children aged 4–10 years who completed one MRI session. Inclusion criteria were lack of contraindications to MRI scanning, typical development, and at least one parent fluent in English. Exclusion criteria were diagnosis of psychiatric or neurologic illness, developmental disorders, inability to undergo MRI scanning, or birthing parents with medical conditions or significant illness during pregnancy. Participants with incomplete MPnRAGE acquisition were excluded from the study, leading to a total of 61 sessions across 43 participants (participant flowchart provided in Supplementary Fig. S1). Experimental protocols were approved by the University of Wisconsin-Madison Human Subjects Institutional Review Board and written informed consent was provided by the parent or primary caregiver for each child.

**Table 1.** Sample demographic characteristics.

Variables	Sample
**Number of subjects**	43
**Mean Age at Scan (years)**	4.4
Range	0.25–11.0
Standard deviation	3.5 years
**Child Sex**	Male n = 27; female n = 16
**Child Race (incl. multiple)** White	26
Asian/Pacific Islander	4
American Indian/AK Native	0
Black/African American	3
Other	0
Not provided	10
Child ethnicity	
Hispanic/Latino	1
Not Hispanic/Latino	37
Not provided	5
**Mean Mat. Education (years)**	14.8
Range	3-21
Standard deviation	5.1
N Not provided	7
**Median Family Income Bracket**	$100,0001–$150,000
Range	($20,001–$30,000) – (>$200,000)
N Not provided	2
**Mean ADI National Percentile**	35.7
Range	12-78
Standard deviation	14.4

### 2.2 Measures

#### 2.2.1 Area deprivation index

ADI United States national percentile was calculated for each participant based on their primary address at the time of recruitment using the 2022 Neighborhood Atlas online tool (Kind & Buckingham, 2018; University of Wisconsin School of Medicine Public Health, n.d.). ADI is a composite measure of neighborhood disadvantage, derived from 17 census-based variables that reflect education, income, employment, housing quality, and household characteristics. These variables are weighted and combined to generate a national percentile ranking from 1 (least disadvantage) to 100 (most disadvantage), indicating the relative disadvantage faced by individuals within a neighborhood compared with others across the United States. In our sample, ADI national percentiles ranged from 12th to 78th percentile, with a median of 34 (Interquartile Range: 17), mean of 35.7 and a standard deviation of 14.4. None of the infants who completed multiple study visits experienced a change in address during the study timeframe, ensuring that ADI values remained stable for longitudinal analyses.

#### 2.2.2 Neuroimaging

##### 2.2.2.1 MRI data acquisition

Brain myelination was assessed using the longitudinal relaxation rate (R1), as measured by the MPnRAGE pulse sequence (Kecskemeti & Alexander, 2020; Kecskemeti et al., 2016). R1 is sensitive to myelin content and captures developmental changes in white matter microstructure (S. C. L. Deoni et al., 2012; Kühne et al., 2021). This approach allows for quantification of myelin maturation during infancy and early childhood, critical developmental windows. MRI data were acquired using a 3T GE Discovery MR750 scanner (Waukesha, WI) with a 32-channel phased array head coil (Nova Medical, Wilmington, MA). Whole-brain structural imaging was performed using a 3D T1-weighted MPnRAGE sequence with 1 mm isotropic resolution and an inversion-recovery magnetization preparation and three-dimensional radial *k*-space acquisition (Kecskemeti et al., 2016). Acquisition parameters included TR = 4.9 ms, TE = 1.8 ms, and 386 views along the recovery curve, with excitation flip angles of 4° for the first 304 views and 8° for the 82 remaining views. A delay time of TD = 500 ms occurred after the last TR of each gradient-echo block to allow the signal to recover before the next preparation pulse. Scan duration was approximately 8 minutes. All children under 4 years of age were scanned during natural, unsedated sleep, while children 4 years and older were scanned while watching a video of their choice and were instructed to remain still. Retrospective motion correction was performed as described in Kecskemeti et al. (2018) and a multi-pass fitting procedure was used to estimate parametric maps of T1 (1/R1) relaxation times (Kecskemeti & Alexander, 2020). Motion-corrected MPnRAGE T1-weighted images and T1 maps were used in subsequent processing and analysis. Importantly, prior validation work using the same motion-corrected MPnRAGE framework has demonstrated that residual head motion does not significantly influence R1 estimates across nearly all cortical and subcortical regions (see fig. 5 in Kecskemeti et al., 2021). This analysis showed that motion metrics were not significantly associated with regional R1 values after retrospective motion correction, indicating that the MPnRAGE reconstruction and fitting procedure substantially mitigate motion-related bias in quantitative T1/R1 measurements. Accordingly, the R1 estimates used in the present study are expected to withstand typical levels of motion encountered in pediatric imaging.

##### 2.2.2.2 MRI region-of-interest (ROI) analysis

Age and study-specific templates were created from participants’ MPnRAGE T1-weighted images using the “antsMultivariateTemplateConstruction2.sh” script as part of ANTs (Avants et al., 2009). Individual participants’ T1 maps were transformed to the template space using the resulting affine and non-linear transformations. The Johns Hopkins University (JHU) ICBM-DTI-81 template (Hua et al., 2008; Mori et al., 2006; Wakana et al., 2007) was spatially aligned to the population template using ANTs (Avants et al., 2009). The population template aligned JHU ICBM-DTI-81 white matter atlas was then transformed to each subject’s native space by applying the inverse of the spatial transformations estimated in the population- and subject-specific template generation step. Median values for each ROI in the atlas were extracted from each participant’s corresponding native-space T1 map. Median values were used as the median is less sensitive than the mean to voxels with extreme values, including outliers at tissue boundaries (Van Belle et al., 2004). R1 values were then calculated as the inverse of T1 relaxation times, to allow for ease of comparison as R1 is thought to increase with myelination. White matter ROIs selected for analysis included regions where previous work identified associations between ELA and brain MRI metrics in children or adults (Dufford et al., 2020; He et al., 2023; Ku et al., 2024; Lammeyer et al., 2019; McCarthy-Jones et al., 2018; Mitchell et al., 2025; Tendolkar et al., 2017; Ugwu et al., 2015), including the anterior, superior, and posterior aspects of the corona radiata; genu, splenium, and body of the corpus callosum; gyral and hippocampal aspects of the cingulum; anterior limb and retrolenticular limb of the internal capsule; inferior and superior fronto-occipital fasciculus; superior longitudinal fasciculus; and uncinate fasciculus. See Supplementary Figure S2 for a full list and atlas of ROIs.

T-tests were conducted to determine differences in R1 between right-sided and left-sided ROIs. There were no significant (p < .05) differences in R1 in bilateral pairs of ROIs, so values in all ROIs represent bilateral averages of median R1 values.

##### 2.2.2.3 MRI exploratory voxel-based analysis

A white matter mask was generated for the voxel-based analysis using the FMRIB Automated Segmentation Tool (FAST) (Zhang et al., 2001). The mask was then thresholded to a 60% probability and smoothed with a 3 mm Full Width at Half Maximum (FWHM) kernel using fslmaths (Smith et al., 2004). The resulting brain mask was then applied to each subject’s template-registered R1 map. The masked R1 maps were then merged into a 4D subject composite using fslmaths (Smith et al., 2004). See Supplementary Figure S2 for a visualization of the voxel-based analysis image processing and analysis steps.

### 2.3 Statistical analysis

First, we used general linear models and linear mixed effects models to examine bivariate associations between ADI, age, and sex. Results are given in Supplementary Figure S3. Then, we assessed associations between neighborhood disadvantage (ADI national percentiles) and myelin content (R1) in ROIs using linear mixed effects models. Inspection of model diagnostics indicated that the distribution of ADI was skewed and violated standard model assumptions, including non-normal and heteroscedastic residuals, as assessed using the GVLMA package in R (Pena & Slate, 2019). To improve model fit and satisfy linear model assumptions, ADI was square-root (sqrt) transformed prior to its inclusion in all mixed-effects analyses. The square-root transformation was chosen based on a Box-Cox analysis (Atkinson et al., 2021; Supplementary Fig. S4). Based on prior work demonstrating a logarithmic relationship between R1 and age in infants and children (S. C. L. Deoni et al., 2011), age was natural log transformed to better capture non-linear developmental effects. Q-Q plots demonstrating the normality of residuals after transformation of the ADI are included in the Supplementary Figure S4. Initial models included main effects of sqrt(ADI), log(age), and sex (hereafter referred to as ADI, age, and sex), as well as their interaction terms. When higher-order or interaction terms were not statistically significant, nested models were compared by removing non-significant terms and evaluating changes in model fit using Akaike’s Information Criterion (AIC; Akaike, 1973; Bozdogan, 1987) and changes in explained variance (ΔR²). More parsimonious models were retained when model fit was equal or improved.

To determine whether ADI was associated with R1 in ROIs, we conducted a series of linear mixed effects models regressing R1 on ADI. We included within-subject random effects given repeated sessions for some subjects. Age was considered a fixed effect to preserve model simplicity (Barr et al., 2013; Brauer & Curtin, 2018). False discovery rate (FDR) adjustments were used to adjust p-values for multiple comparisons, correcting for the number of ROIs tested (Benjamini & Hochberg, 1995). To ensure our findings were robust to potential inflation of Type 1 Error that can be introduced by linear mixed effects models without random effects (Brauer & Curtin, 2018), we conducted two sensitivity analyses: (1) general linear models restricted to the first datapoint for each subject and (2) models restricted to the younger and older sub-sample of participants. Additionally, we conducted a sensitivity analysis including a composite household income and maternal education variable (income_ed) to assess whether these individual-level factors influenced associations with ADI. The exact model specifications, including fixed and random effects and all sensitivity analyses, are provided in the Supplementary Equations S1–S4.

For exploratory voxel-wise analyses, we constructed a voxel-based linear mixed effects model to investigate associations between ADI and R1 across white matter, again covarying for age and a within-subject random effects covariate. Given that this more exploratory analysis was conducted across the entire brain, we used p < .005 (not FDR-corrected) as our significance threshold. The resulting output maps generated using ANTsR (Avants et al., 2009) showed associations between each covariate and R1, thresholded to p < .005.

## 3 Results

### 3.1 ADI was negatively associated with R1 across white matter ROIs

**Fig. 1.**
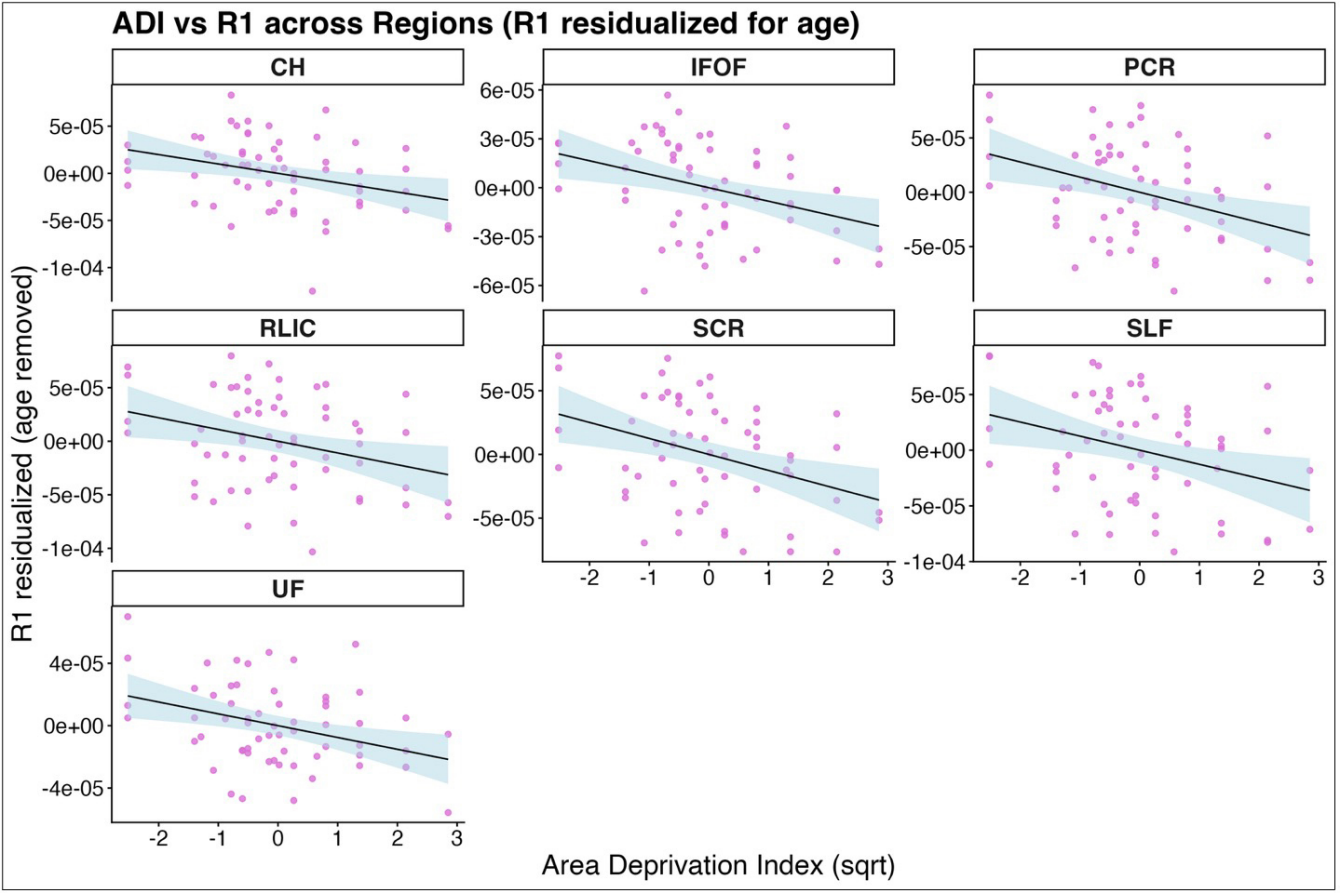
Associations between ADI (square root transformed(sqrt)) and R1 (residualized with respect to log(age)) in significant ROIs: cingulate gyrus–hippocampal aspect (CH), inferior fronto-occipital fasciculus (IFOF), posterior corona radiata (PCR), retrolenticular limb of the internal capsule (RLIC), superior longitudinal fasciculus (SLF), and uncinate fasciculus (UF). The light blue shaded regions represent 95% confidence intervals.

ADI was significantly negatively associated (p_corr_ < .05) with R1 in the following ROIs: the retrolenticular limb of the internal capsule, superior and posterior corona radiata, cingulate gyrus–hippocampal aspect, inferior fronto-occipital fasciculus, superior longitudinal fasciculus, and uncinate fasciculus (Table 2). Effect sizes for these ADI-R1 associations were small, with ΔR^2^ values ranging from .006 to .013. Figure 1 illustrates relationships between ADI and R1 in all significant ROIs (plots for non-significant ROIs available in Supplementary Fig. S5). Age was significantly positively associated with R1 in all models (p_corr_ < .05), and there were no significant age-by-ADI interactions. Sex was also nonsignificant in all models (p_corr_ > .05). Full model summaries for significant ROIs are provided in Table 2.

**Table 2.** ROIs where ADI was significantly negatively associated with R1.

ROI	Standardized β (ADI)	Standardized β 95% CI (ADI)	t-statistic (ADI)	p_corr_ (ADI)	ΔR²
Cingulate–Hippocampal Aspect	-0.12	[-0.20, -0.45]	-2.74	.025	.010
Inferior Fronto-Occipital Fasciculus	-0.10	[-0.16, -0.03]	-3.06	.023	.007
Posterior Corona Radiata	-0.12	[-0.20, -0.04]	-3.16	.023	.012
Retrolenticular Limb of the Internal Capsule	-0.11	[-0.19, -0.02]	-2.58	.032	.009
Superior Corona Radiata	-0.11	[-0.19, -0.03]	-2.87	.023	.010
Superior Longitudinal Fasciculus	-0.11	[-0.18, -0.03]	-2.84	.023	.008
Uncinate Fasciculus	-0.12	[-0.21, -0.04]	-2.89	.023	.013

Standardized β values represent the change in R1 (in standard deviations) associated with a 1 standard deviation increase in square-root–transformed ADI. Negative coefficients indicate lower R1 values with increasing neighborhood deprivation. The table reports standardized β estimates with 95% confidence intervals, corresponding t-statistics, and false discovery rate-corrected p-values across ROIs. ΔR² reflects the increase in marginal variance explained by adding ADI to the age-only model. Significant associations were observed in several projection and association tracts, including the cingulate–hippocampal aspect, inferior fronto-occipital fasciculus, posterior and superior corona radiata, retrolenticular limb of the internal capsule, superior longitudinal fasciculus, and uncinate fasciculus.

Sensitivity analyses conducted separately within the younger infant sub-sample (37 sessions across 19 children) and the older child sub-sample (24 children, 1 session each) indicated that associations between higher ADI and lower R1 were significant after FDR correction in the younger cohort but not in the older cohort. To further evaluate the strength of our findings and potential inflation of Type 1 error in the linear mixed effects models without a within-subject age random slope, we repeated the analysis using only the 43 first-session datapoints (Supplementary Table S2). Results were generally consistent across ROIs; the exception was the uncinate fasciculus (p_corr_ = .117), which may reflect either reduced power due to the smaller sample or instability of findings in this ROI. Because the overall pattern of associations was preserved in the first-session analysis, these secondary analyses are presented but are not discussed further in the main text.

### 3.2 Exploratory voxel-based analysis reveals associations between ADI and white matter R1

**Fig. 2.**
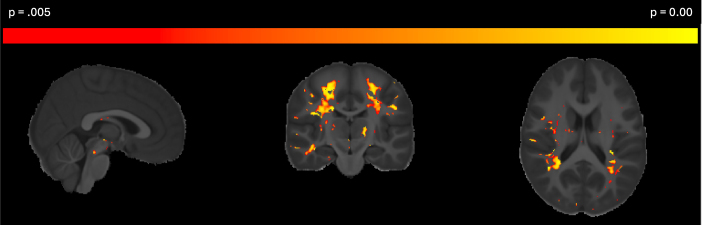
Heatmap of white matter voxels where ADI was negatively associated with R1, p < .005.

Given the significant associations between ADI on R1 in our ROI analysis, we next conducted an exploratory voxel-based analysis to examine the extent of associations throughout the white matter. We found associations between ADI and R1 as indicated by the significant clusters shown in Figure 2. All t-statistics in significant clusters were negative, indicating a negative association between ADI and R1 (Supplementary Fig. S6). In addition to the regions identified in the ROI analysis, significant clusters were identified in the bilateral posterior thalamic radiations. As in the ROI analysis, the age-by-ADI interaction and sex were removed to improve model fit assessed by ΔR^2^ and AIC.

## 4 Discussion

We evaluated how early life neighborhood disadvantage is associated with white matter myelination across infancy and childhood. Using R1, a quantitative MRI marker sensitive to myelin content, we identified associations between greater neighborhood disadvantage (higher ADI national percentile) and lower white matter myelination (lower R1). ROI-based analyses demonstrated consistent links across white matter regions, while exploratory voxel-wise analyses suggested that these associations may extend across the white matter. Together, these findings indicate that exposure to neighborhood-level disadvantage is associated with lower white matter maturity early in life, underscoring the salience of contextual, macrostructural factors for neurodevelopment.

### 4.1 Neighborhood disadvantage and early myelin development

Our findings demonstrate associations between neighborhood disadvantage and lower myelin-sensitive R1 values across the brain’s white matter. Prior neuroimaging studies examining early life adversity and socioeconomic disadvantage have reported differences in white matter microstructure, including lower fractional anisotropy and altered diffusivity in tracts supporting cognitive and emotional processing such as the corpus callosum, uncinate fasciculus, and cingulum (e.g., Ku et al., 2024; McCarthy-Jones et al., 2018; Ugwu et al., 2015). Our findings extend this work by suggesting that neighborhood-level disadvantage may be associated with alterations in white matter maturation during development. Myelination is a fundamental neurobiological process supporting efficient neural communication, network integration, and cognitive and behavioral development (Beaulieu et al., 2020; Corrigan et al., 2022; Fornari et al., 2007). R1 provides a sensitive, developmentally appropriate marker of white matter maturation that increases with myelin content and has been widely used to characterize normative and atypical brain development (S. C. L. Deoni, 2010; S. C. L. Deoni & Dean, 2021). By quantifying R1 across a broad pediatric age range, our study captures meaningful variability in white matter maturation during periods of heightened neuroplasticity and extends prior work by examining associations between neighborhood disadvantage and a quantitative measure sensitive to myelin content. The observed associations between neighborhood disadvantage and lower R1 align with the literature linking adversity to differences in white matter development, while providing additional insight regarding macrostructural-level disadvantage and potential myelin-related processes that may contribute to these patterns.

### 4.2 Regional specificity of ADI–R1 relationships

The regions identified in our ROI analyses play critical roles in executive function, emotion regulation, learning, and adaptive behavior across development (Caldinelli et al., 2017; Frye et al., 2010; Herbet et al., 2017; Lövblad et al., 2014; McLaughlin et al., 2018; Olson et al., 2015; Rose et al., 2007; Stave et al., 2017; Vandecruys et al., 2024). The convergence of associations across multiple ROIs suggests that neighborhood disadvantage may be related to white matter systems supporting broad functional networks, rather than targeting a single isolated pathway. White matter circuitry potentially influenced by adversity based on our analysis includes fronto-limbic regions (cingulum and uncinate fasciculus), which are involved in socioemotional functioning (Hendrikse et al., 2024) and long-range white matter circuitry (corona radiata, internal capsule, inferior and superior fronto-occipital fasciculi, and superior longitudinal fasciculus), which supports diverse executive functions (Ribeiro et al., 2024). Importantly, the consistency between ROI-based and voxel-wise findings strengthens confidence in these associations and highlights the utility of combining hypothesis-driven and exploratory neuroimaging approaches.

### 4.3 Potential mechanisms and developmental implications

The observed associations between ADI and R1 may reflect indirect and interacting pathways, including access to health care, community support, caregiver stress, nutrition, or environmental exposures (Chilton et al., 2007; Cowell et al., 2025; Evans, 2004, p. 200; Luby et al., 2013; Miguel et al., 2019). Because ADI captures contextual neighborhood characteristics rather than direct measures of individual adversity exposure, these associations likely reflect a constellation of co-occurring environmental factors rather than a single causal mechanism. Importantly, sensitivity analyses including a composite measure of household income and maternal education (income_ed) did not meaningfully alter the associations between ADI and R1 across ROIs, nor did they improve model fit. This suggests that the observed associations may relate more strongly to neighborhood-level contextual factors than to concurrent individual-level socioeconomic differences in this sample.

At the same time, prior work has suggested that altered white matter maturational trajectories, particularly in fronto-limbic structures, may be suggestive of neurodevelopmental plasticity, wherein changes to the timing or extent of brain maturation may prioritize functions necessary for coping with stress or environmental unpredictability (Cook et al., 2024; Lean et al., 2022). Consistent with this perspective, sub-sample sensitivity analyses indicated that associations between neighborhood disadvantage and myelination were strongest in the younger infant cohort, a developmental period characterized by rapid white matter growth. In the older child cohort, which included fewer participants and only a single session per child, these associations did not survive multiple comparisons correction. As ADI-by-age interactions were not significant in full-sample analyses, subgroup differences may reflect reduced statistical power rather than true developmental specificity. Several alternative explanations should also be considered. First, early infancy represents a period of particularly rapid and experience-dependent myelination, during which white matter microstructure may be especially sensitive to environmental adversity; by early childhood, developmental trajectories may have partially stabilized or diverged in ways that obscure a linear ADI effect. Second, demographic differences between subsamples due to differing recruitment strategies are another possible contributor, though ADI, maternal education, and family income did not significantly differ between groups (Supplementary Table S1). Differences in developmental timing, or some combination of these factors, may also play a role, and findings should, therefore, be interpreted cautiously. It remains possible that the association between neighborhood disadvantage and white matter myelination is concentrated during infancy and attenuates over time as children accumulate protective experiences, such as supportive caregiving or early educational enrichment. Future longitudinal studies are needed to clarify whether these associations persist, diminish, or are moderated by such factors across childhood.

Together, our findings highlight the importance of considering developmental timing and sample structure when examining associations between environmental disadvantage and neurodevelopment. Our findings are broadly consistent with prior work suggesting that environmental disadvantage may be associated with differences in early neural architecture that are strongest in infants and could have implications for later developmental outcomes. A key strength of the present study is the integration of ROI and voxel-wise analyses within a sample spanning infancy through middle childhood. This approach enabled us to characterize associations between neighborhood disadvantage and white matter maturation across a wide developmental window, providing novel insight into how structural inequities may become biologically embedded early in life.

### 4.4 Limitations

There are also significant limitations to our constructs that should be acknowledged, even as they highlight important strengths of the current approach. While ADI captures multiple neighborhood-level features relevant to health and development, it does not directly index experiences of racial discrimination or all forms of structural inequality, nor does it account for children’s exposure to environments beyond their primary residence (Heard-Garris et al., 2021; Riley, 2018; Ursache et al., 2021; Zenk et al., 2011). Additionally, although R1 is a sensitive marker of white matter maturation, it is not fully specific to myelin and may also reflect other microstructural properties such as water or iron content (S. C. L. Deoni, 2010; S. C. L. Deoni & Dean, 2021). Future studies incorporating complementary quantitative MRI methods will help refine biological specificity. More broadly, our sample size, age distribution, and demographic composition (with significantly lower average neighborhood disadvantage than the national average) limit power to detect small effects, developmental interactions, and subgroup differences. Our sample of 61 timepoints across 43 children was powered to detect medium to large effects (ηp² = 0.15), limiting sensitivity to smaller associations. Furthermore, significant associations between neighborhood disadvantage and white matter myelination survived multiple comparisons correction only in the infant subsample. This may reflect a sensitive period during which rapidly developing white matter is particularly vulnerable to environmental adversity, though reduced statistical power in the smaller older subsample cannot be ruled out as an alternative explanation. The modest effect sizes observed may reflect protective factors in this cohort, the multifactorial nature of early life disadvantage, and heterogeneity in children’s experiences (Lopez et al., 2021), but are consistent with effect sizes commonly reported in neurodevelopmental research (Bakker et al., 2019; Schäfer & Schwarz, 2019; Szucs & Ioannidis, 2017). A further limitation is the absence of concurrent behavioral or developmental outcome measures, which prevents direct assessment of how the observed differences in R1 relate to cognitive, socioemotional, or motor function in this cohort. Future studies should include clinically relevant developmental outcome measures to assess the functional significance of observed differences in white matter myelination. Finally, we note that our primary models included only age, sex, and the ADI-by-age interaction as covariates. Gestational age at birth was available and included in sensitivity analyses, which did not materially change the pattern of significant results. Other potentially important confounds, such as birth weight, prenatal exposures (e.g., maternal substance use or stress), and postnatal environmental factors, were not available in this cohort. Although these factors may plausibly covary with both ADI and brain myelination, the present associations remain informative as an initial characterization of neighborhood-level influences on early white matter development. Future studies with larger, more diverse cohorts and richer perinatal and postnatal data will be necessary to further disentangle the contributions of neighborhood disadvantage from these individual-level exposures and to identify protective factors that may buffer risk.

### 4.5 Future directions

In addition to addressing the limitations of our current work, future endeavors should evaluate how protective factors may buffer associations between disadvantage and brain development. Children exposed to disadvantage may also access protective environmental resources such as responsive caregiving, emotional support, modeled resilience, community cohesion, or individual traits such as emotion regulation and cognitive flexibility (Béné et al., 2014; Judge, 2005; Vanderbilt-Adriance & Shaw, 2008). Nonetheless, disadvantage and resource limitations often co-occur and interact in non-linear ways, necessitating dimensional approaches to studying ELA, particularly when investigating its neurobiological correlates (DeJoseph et al., 2021, 2024). Examining how these factors operate across developmental stages could clarify how different factors shape children’s vulnerability to adversity, informing future interventions and policy initiatives.

## 5 Conclusion

Our findings highlight associations between neighborhood-level disadvantage and myelin maturation, which were stronger in the infant subsample and may reflect differences in environmental exposures or adaptive neurodevelopmental responses in the context of adversity. Future work should extend these results to larger, nationally representative populations to further characterize how neighborhood disadvantage relates to brain development and developmental performance during infancy and childhood. Additionally, future work should investigate protective factors and sources of resilience that may alter developmental trajectories for children growing up in disadvantaged environments.

## Supplementary Material

Supplementary Material

## Data Availability

The data analyzed for this study are available upon request from the corresponding author. All scripts were created in RStudio and will be shared upon request to the corresponding author.
