# Supplementary material for: Neighborhood disadvantage and brain myelination: Insights from infancy to childhood
Source: Imaging Neurosci (Camb). 2026 Jun 25;4:IMAG.a.1288. doi: 10.1162/IMAG.a.1288 (PMC13308800; doi:10.1162/IMAG.a.1288)

Table S1

Sample demographics for sub-samples (infant and child). Demographic variables are compared between younger (< 3.8 years) and older (≥ 3.8 years) subgroups; Age at scan, maternal education, and ADI percentile were compared using Welch's t-tests, family income bracket was compared using a Mann-Whitney U test, and sex was compared using chi-square tests.

Variables	Infant Sample	Child Sample	P-value
**Number of Subjects**	19 (37 Sessions)	24	
**Mean Age at Scan (years)**	1.36	7.1	0.000
Range	0.25 - 2.85	4.0 - 11.0	
Standard Deviation	0.72	2.1	
**Child Sex**	Male n = 11, Female n = 8	Male n = 16; Female n = 8	0.785
**Child Race (incl. multiple)**			
White	11	18	
Asian / Pacific Islander	2	2	
American Indian / AK Native	0	0	
Black / African American	1	2	
Other	0	0	
Not provided	5	4	
**Child Ethnicity**			
Hispanic/Latino	1	0	
Not Hispanic/Latino	18	19	
Not provided	0	5	
**Mean Mat. Education (years)**	18.53	19.4	0.090
Range	15-21	18-21	
Standard Deviation	1.7	1.2	
N Not provided	2	6	
**Median Family Income Bracket**	$90,001-$100,000	$100,001-$150,000	0.211
Range	($20,001 -$30,000) – (>$200,000)	($20,001 -$30,000) – (>$200,000)	
N Not provided	2	0	
**Mean ADI National Percentile**	39.4	32.8	0.155
Range	12-78	12-66	
Standard Deviation	16.2	12.3	

Figure S1

Flowchart of subjects and data missingness.

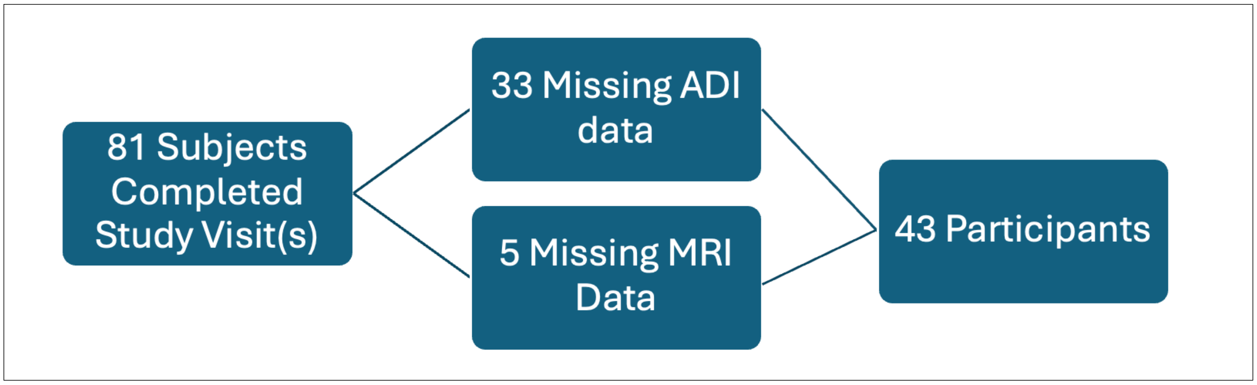

Figure S2

A) Regions of Interest for Analysis. Regions are listed as they appear in the slice above each column, organized from most superior to most inferior structures. Colors correspond to displayed regions. Regions labeled in white are not visible in the illustrated slices. B) Overview of voxel-based analysis preprocessing steps.

A)

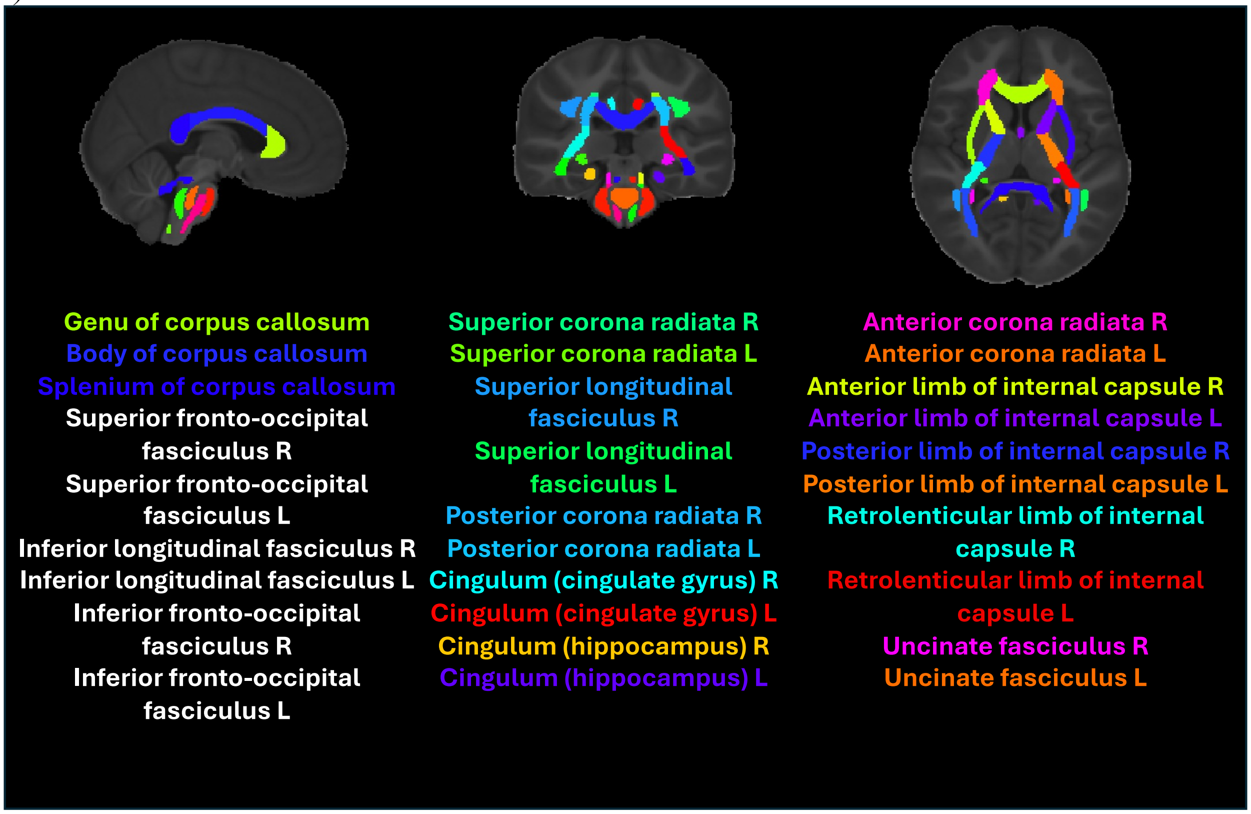

B)

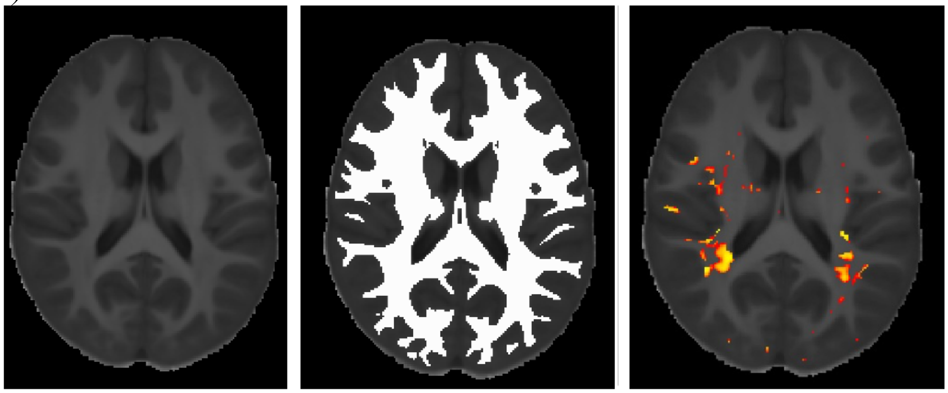

R1 map, smoothed to 3 mm Full Width at Half Maximum Kernel

Reference white matter mask

LME within all white matter mask voxels ($p < .005$)

Figure S3

Distributions and associations among key variables (not transformed). Age at scan and ADI were negatively associated (linear mixed-effects model: β = –0.09, p = 0.012). No significant associations were observed between ADI and sex (linear model) or between age and sex (linear mixed-effects model). Panels illustrate variable distributions and pairwise associations, generated using the GGally package in R (Schloerke et al., 2010). ***Top row:*** *Density plot of age at scan.* ***Middle row:*** *Association between age and sex (left) and sex distribution (right).* ***Bottom row:*** *Association between age and ADI (left), Association between sex and ADI (middle), and density plot of ADI (right).*

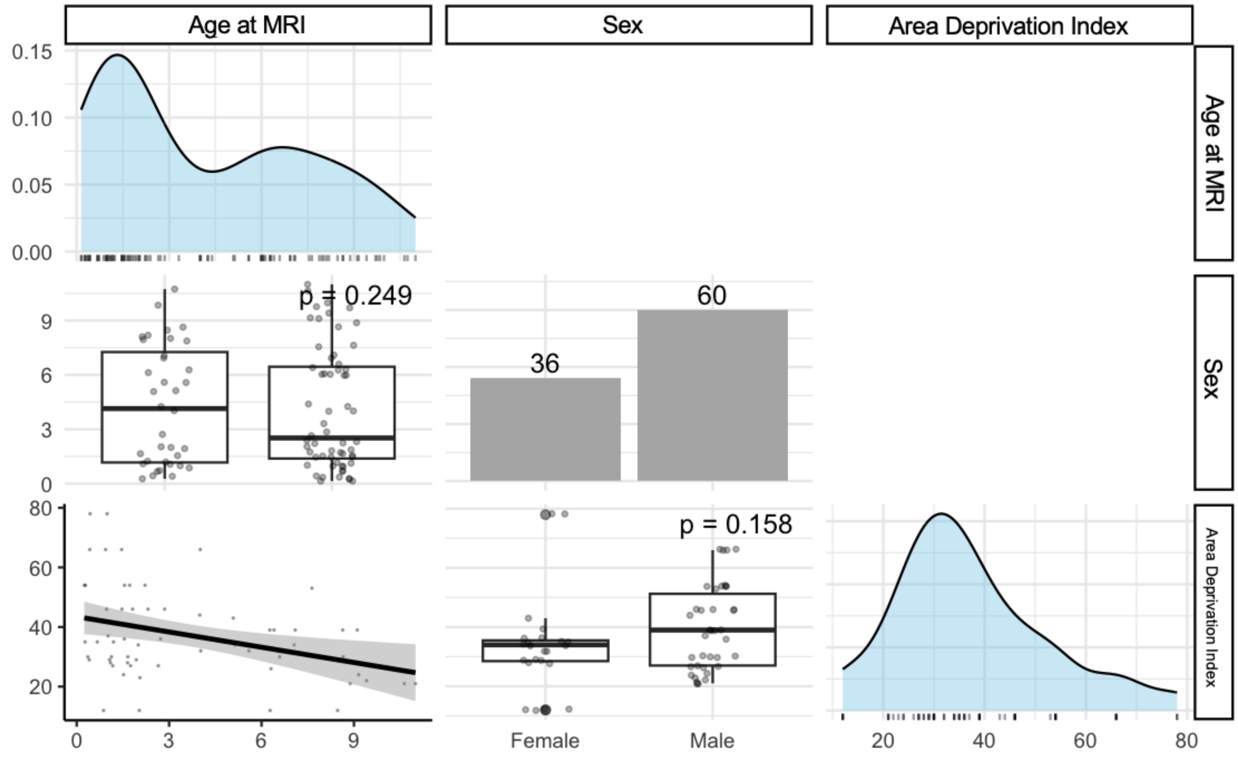

Figure S4

(A) Histogram of raw ADI national percentiles, illustrating the skew of the distribution. (B) Histogram of square-root transformed ADI national percentiles. (C) The square-root transformation was applied to improve normality based on a Box-Cox analysis (λ ≈ 0.34; rounded to square root λ = 0.5 for interpretability). Transformation choice was guided by a linear model of ADI as a function of age at scan and sex (lm(ADI.national ~ AgeAtScan + sex_numeric, data = new_dataset)). The square-root transformation was used for subsequent parametric analyses to meet model assumptions. (D) Q-Q plots showing the normality of model residuals after square root transformation of ADI.

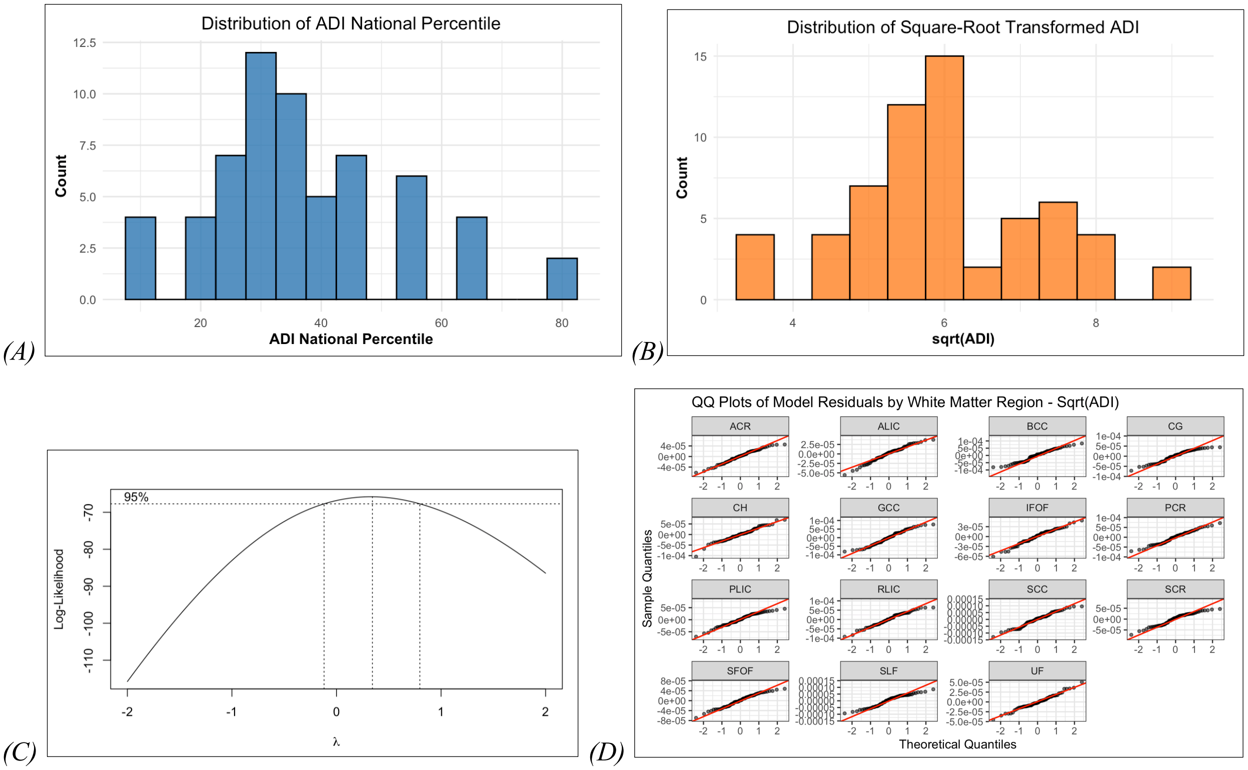

Supplementary Model Specification

For each region of interest (ROI), associations between neighborhood deprivation and myelin content were evaluated using linear mixed effects models of the following form:

$$R1_{ij} = \beta_0 + \beta_1\sqrt{ADI}_{ij} + \beta_2\ln{(Age_{ij})} + u_{0i} + \epsilon_{ij} \qquad \text{(S1)}$$

where $R1_{ij}$ represents the longitudinal relaxation rate (1/T1) for subject i at session j, $\sqrt{ADI}_{ij}$ is the square-root transformed Area Deprivation Index (national percentile), and $\ln{(Age_{ij})}$ is the natural log–transformed age at scan. β_0 denotes the intercept, β_1 and β_2 represent fixed effects for ADI and age, respectively, u_{0i} represents the subject-specific random intercept accounting for repeated observations, and ϵ_{ij} represents the residual error term.

Because repeated observations occurred primarily in the infant subgroup and the number of longitudinal observations per subject was limited, the primary models included a random intercept for subject but not random slopes. To evaluate the robustness of this specification, we conducted two sensitivity analyses. First, we tested models including a random slope for age within subject:

$$R1_{ij} = \beta_0 + \beta_1\sqrt{ADI}_{ij} + \beta_2\ln{(Age_{ij})} + u_{0i} + u_{1i}\ln{(Age_{ij})} + \epsilon_{ij} \qquad \text{(S2)}$$

where u_{1i} represents the subject-specific slope for age. These models did not materially change the estimated association between ADI and R1 across ROIs and frequently produced singular fits due to the limited number of repeated observations per subject.

Second, to confirm that findings were not driven by repeated measurements, we conducted a sensitivity analysis restricted to the first scan per subject, estimating the following general linear model:

$$R1_i = \beta_0 + \beta_1\sqrt{ADI}_i + \beta_2\ln{(Age_i)} + \epsilon_i \qquad \text{(S3)}$$

Additionally, to examine whether household income and maternal education influenced these associations, we conducted an additional sensitivity analysis including a composite income/education variable (income_ed) as a covariate in the models:

$$R1_{ij} = \beta_0 + \beta_1\sqrt{ADI}_{ij} + \beta_2\ln{(Age_{ij})} + \beta_3 income\_ed_{ij} + u_{0i} + \epsilon_{ij} \qquad \text{(S4)}$$

Across all ROIs, including income_ed did not improve model fit (AIC was higher across models upon inclusion of income_ed) or alter the estimated associations between ADI and R1.

Finally, sub-sample analyses were conducted using the same linear mixed-effects model framework as the primary analyses (Equations S1–S4), restricted to the younger infant cohort or the older child cohort. Results confirmed that ADI-R1 associations were present in the younger cohort but not in the older cohort.

Results from all sensitivity analyses were consistent with the primary mixed-effects models. False discovery rate (FDR) correction was applied across ROI analyses to account for multiple comparisons.

Figure S5

Scatterplots of ADI vs R1 (residualized with respect to age) in all regions of interest. Asterix (*) indicate significant regions.

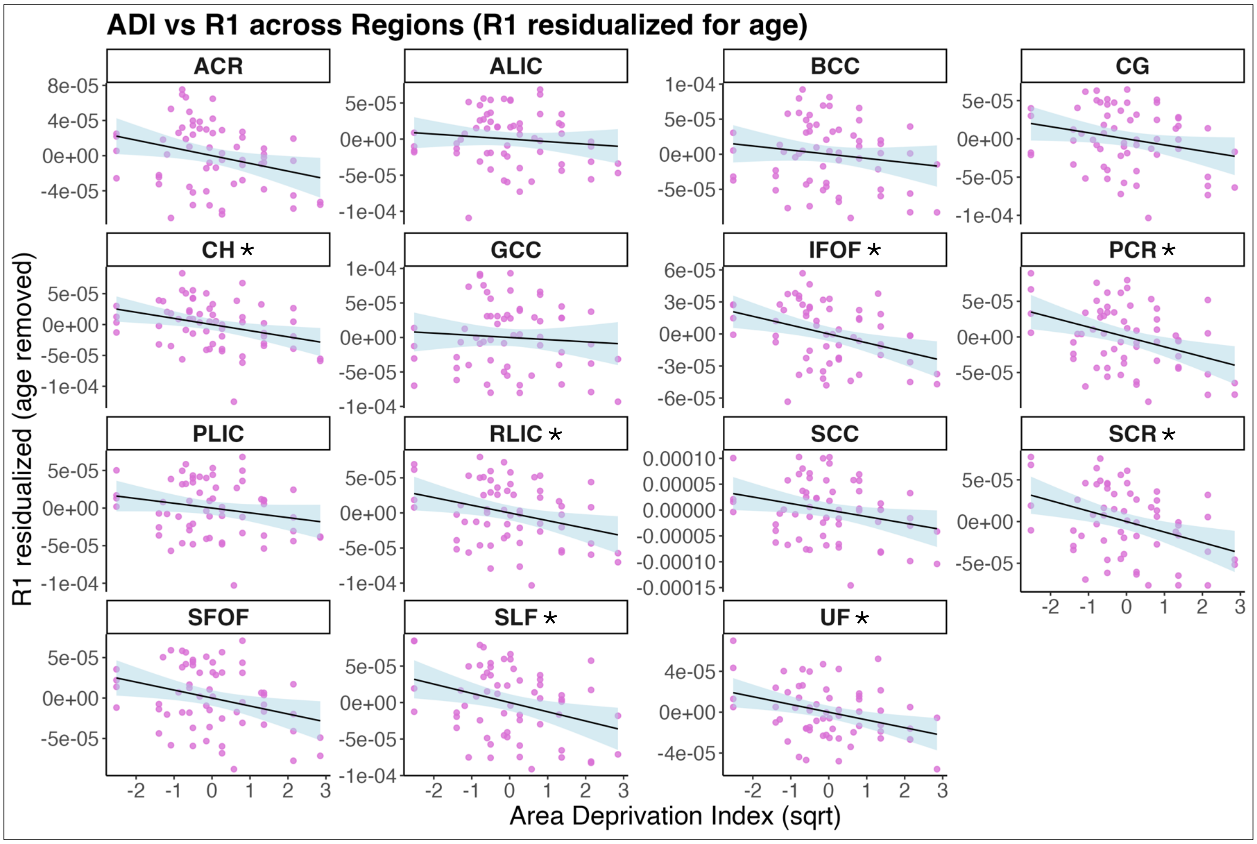

Table S2

Regions of interest where the ADI is significantly negatively associated with R1 ($p_{corr} < .05$), subsample including only session 1 data (n= 41). Bolded text indicates regions where the ADI was significantly negatively associated with R1 in the full sample (n = 61) but not the session 1 subsample.

**Region of Interest**	**t-statistic (ADI)**	**p_{corr} (ADI)**	**ΔR^2**
Cingulate - Hippocampal Aspect	-2.53	.034	.009
Inferior Fronto-Occipital Fasciculus	-2.40	.035	.005
Posterior Corona Radiata	-2.91	.030	.010
Posterior Limb of the Internal Capsule	-2.14	.058	.007
Retrolenticular Limb of the Internal Capsule	-2.56	.034	.010
Splenium of the Corpus Callosum	-2.49	.034	.011
Superior Corona Radiata	-3.06	.030	.011
Superior Fronto-Occipital Fasciculus	-2.47	.034	.007
Superior Longitudinal Fasciculus	-2.97	.013	.011
**Uncinate Fasciculus**	**-1.68**	**.12**	**.003**

Figure S6

T-statistics for voxel-based analysis (hypothesis 2). All t-statistics in significant clusters were negative, ranging from -6.24 to 0.

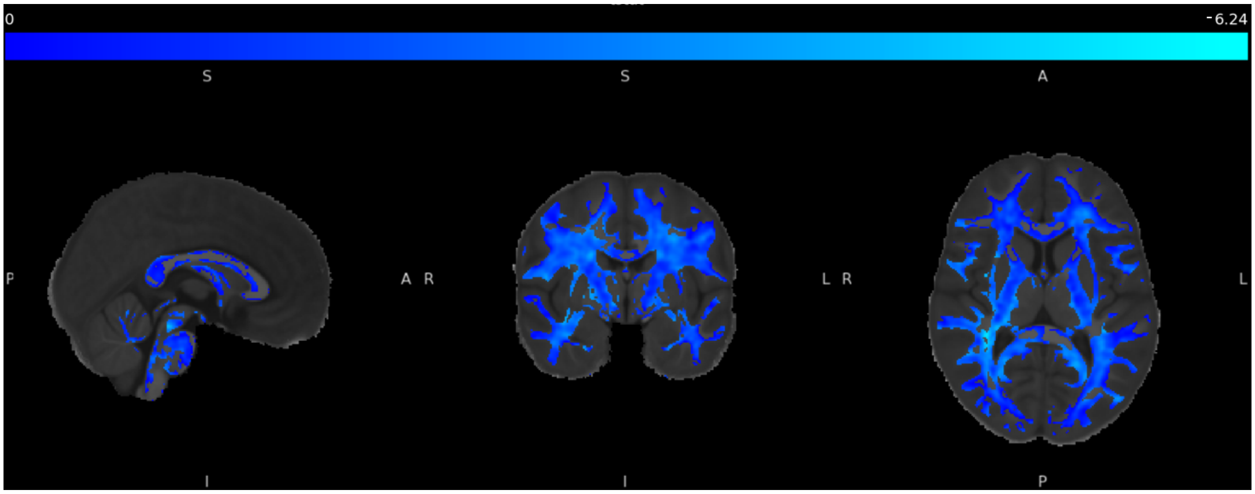

Supplement: Supplementary Material [file IMAG.a.1288_supp.pdf]
